# Early Response of the *Populus nigra* L. × *P. maximowiczii* Hybrid to Soil Enrichment with Metals

**DOI:** 10.3390/ijms252312520

**Published:** 2024-11-21

**Authors:** Monika Gąsecka, Kinga Drzewiecka, Zuzanna Magdziak, Włodzimierz Krzesiński, Jędrzej Proch, Przemysław Niedzielski

**Affiliations:** 1 Department of Chemistry, Poznań University of Life Sciences, Wojska Polskiego 75, 60-625 Poznan, Poland; zuzanna.magdziak@up.poznan.pl; 2 Department of Vegetable Crops, Poznań University of Life Sciences, Dąbrowskiego 159, 60-594 Poznan, Poland; wlodzimierz.krzesinski@up.poznan.pl; 3 Department of Analytical Chemistry, Faculty of Chemistry, Adam Mickiewicz University, Uniwersytetu Poznańskiego 8, 61-614 Poznan, Poland; jedrzej.proch@amu.edu.pl (J.P.); pnied@amu.edu.pl (P.N.)

**Keywords:** low-molecular-weight organic acids, phenolic compounds, metal accumulation, physiological parameters, phytoremediation, poplar

## Abstract

This study aimed to investigate the response of *Populus nigra* L. × *Populus maximowiczii* to the addition of selected metals in soil. Rooted cuttings were planted in pots containing soil enriched with equimolar concentrations of Pb, Zn, Al, Ni, and Cu (500 mL of 4 mM solutions of single metal salts: (Pb(NO_3_)_2_; Zn(NO_3_)_2_ × 6H_2_O; Al(NO_3_)_3_ × 9H_2_O; Ni(NO_3_)_2_ × 6H_2_O; or Cu(NO_3_)_2_ × 3H_2_O). Growth parameters, metal accumulation, and physiological and biochemical parameters were assessed after four weeks of cultivation, simulating early response conditions. The results showed diverse metal accumulation in poplar organs, along with an increase in biomass and minor changes in gas exchange parameters or chlorophyll fluorescence. Among low-molecular-weight organic acids, citric and succinic acids were dominant in the rhizosphere, and roots with malonic acid were also present in the shoots. Only *p*-coumaric acid was found in the phenolic profile of the roots. The shoots contained both phenolic acids and flavonoids, and their profile was diversely modified by particular metals. Sucrose and fructose content increased in shoots that underwent metal treatments, with glucose increasing only in Cu and Al treatments. Principal component analysis (PCA) revealed variations induced by metal treatments across all parameters. Responses to Pb and Zn were partially similar, while Cu, Ni, or Al triggered distinct reactions. The results indicate the adaptation of *P. nigra* L. × *P. maximowiczii* to soil containing elevated levels of metals, along with potential for soil remediation and metal removal. However, further studies are needed to evaluate the effect of differences in early responses to particular metals on plant conditions from a long-term perspective.

## 1. Introduction

Metals and their complexes are natural components of the lithosphere. Certain metals support plant growth, physiology, and metabolism as essential mineral nutrients but can also be toxic at excessive concentrations [1,2]. For example, copper (Cu) is vital as a cofactor in metalloproteins, yet excess Cu triggers oxidative stress by increasing reactive oxygen species (ROS) [3]. Zinc (Zn), a trace element in many enzymes, plays roles in seed germination, photosynthesis, and stress resistance; however, Zn excess disrupts growth and leads to cell death [1,4,5,6]. Nickel (Ni) is necessary for urease in nitrogen metabolism, but excess Ni impairs leaf development [7]. Aluminum (Al), while not essential, can promote growth and pathogen resistance and also cause oxidative stress and cellular changes [8]. These metals are toxic at high concentrations, causing physiological and metabolic changes in plants [9,10,11,12]. Oxidative stress induced by redox-active and -inactive metals leads to ROS production, which damages plant cells [1,13]. Redox-active metals form free radicals through the Haber–Weiss and Fenton reactions, while redox-inactive metals disrupt the electron transport chain [1,14].

Natural processes and intensive anthropogenic activities have led to elevated and even toxic levels of certain metals in the soil. As a result, effective detoxification methods are being sought to counteract the negative effects of metal accumulation. Phytoremediation is one such method, offering an ecological and economical solution; however, its limitations include low effectiveness and the lack of efficient ways to utilize contaminated biomass [15]. Research has revealed the possibility of using some species of trees for phytoremediation (dendroremediation) purposes (*Populus*, *Salix*, *Pinus*, *Betula*, *Acer*, *Ulmus*) [16,17,18,19,20]. Fast growth, deep root systems, ability to thrive in nutrient-poor soil, and resistance to metal contamination make these trees promising candidates for dendroremediation [21]. Poplar (*Populus* L.), a genus of fast-growing trees in the willow family (*Salicaceae*), includes around 30 species found mainly in the Northern Hemisphere [22]. *Populus* species and hybrids are excellent candidates for dendroremediation [16,23,24,25]. Japanese poplar hybrid (*Populus nigra* L. × *Populus maximowiczii*), for example, demonstrated high tolerance to metals in a hydroponic screening study by Migeon et al. (2012) [26]. However, physiological and biochemical parameters beyond the biomass assessment have yet to be investigated.

The aim of this study was to investigate the impact of metal (Pb, Zn, Al, Ni, or Cu) presence in soil on growth and metal accumulation abilities of *P. nigra* L. × *P. maximowiczii.* Metals were added to the soil in the form of nitrates in equimolar concentration to enable the comparison analysis between metals. The level of soil enrichment was set based on permissible metal concentrations in agricultural soils (lower guideline values) [27] and criteria for contamination of surface soil layers with heavy metals used in Poland (II–V level of contamination) [28]. Consequent differences resulted from molecular weight values of applied metals. Additionally, the study aimed to assess differences in plant physiological and biochemical reactions to metals (photosynthesis, gas exchange gas chlorophyll fluorescence, the content of organic acids, phenolic compounds, and sugars) to verify the hypothesis that moderate soil contamination induces distinct mechanisms of plant acclimatization and enhances plant growth as an early response. A principal component analysis (PCA) was applied for a comprehensive evaluation of the overall response of the hybrid to various metals.

## 2. Results

### 2.1. Biomass Characteristics

**Figure 1:**
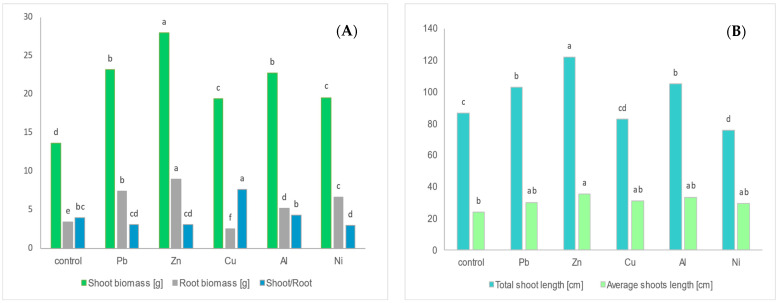
Characterization of *Populus nigra* L. *× P. maximowiczii* hybrid biomass (**A**) and shoots length (**B**) after 4 weeks of growth in soil enriched with selected metal salts. n = 3; identical superscripts (a, b, c, …) denote non-significant differences between means according to the post hoc Newman–Keuls test.

The biomass of particular organs showed significant variation between treatments. After adding metals to the substrate, stimulation of shoot growth (the highest for Zn, followed by Pb~Al > Cu~Ni) and root growth (the highest for Zn, followed by Pb > Ni > Al > Cu) was observed (Figure 1A). However, the shoots and roots biomass ratio was highest for Cu and lowest for Ni treatment. The average number of shoots for Pb-, Zn-, Cu-, and Al-treated plants (2.83–3.67) was similar to the control; however, it was significantly lower for Ni-treated plants (2.67). The total shoot length decreased in the following order: Zn > Al~Pb > control > Cu > Ni (Figure 1B). The average shoot length for the Zn treatment was also higher than that for the control.

### 2.2. Content of Applied Metals in Plant Organs

In roots, the increase in the content of applied metal was observed in the following order: Pb > Ni > Cu > Al, with increases of approximately 20-fold, 9-fold, and 6-fold compared to the control plants, respectively (Table 1).

The content of Pb was below the detection limit in rods of control, Zn-, Al-, and Ni-treated plants, and its content was the highest for Pb-treated plants. The Zn, Cu, and Ni treatments significantly increased the metal content in rods. The content of Al in rods was similar for all variants.

Pb was exclusively detected in the shoots of Pb-treated plants. Zn, Cu, Al, and Ni content in shoots increased in the metal-treated plants compared to the control and remaining treatments.

### 2.3. The Effect of Metal Treatments on Physiological Parameters

The Pb treatment decreased the net photosynthetic rate (A), while Zn, Cu, and Al treatments increased it (Table 2) as measured on young, fully expanded leaves. Transpiration (E) for Cu treatment was lower than in control. In contrast, Zn-treatment decreased stomatal conductance (g_s_). The fluorescence light curves (LCs) (Table 2) showed that Zn treatment decreased electron transport at photosynthetic photon flux density (PPFDs) of 500 and 1000 μmol m^−2^ s^−1^, while Al treatment decreased electron transport at PPFDs of 100, 200, 300, and 500 μmol m^−2^ s^−1^ (Table 2). The LC slope at low PPFD values (parameter α) depended on the metal, and the addition of Zn, Cu, and Al decreased this slope.

The parameters of the OJIP test did not depend on Pb and Al treatments (Table 2). Zn treatment caused an increase in the density of reactive centers (RC) on absorbed energy by chlorophyll (RC/ABS), energy absorption by the reactive center (ABS/RC), capture of electrons excited by RC (TRo/RC), and total dissipation of energy not captured by RC in the form of heat, fluorescence, and transfer to other systems (DIo/RC). Cu- and Ni-treated plants showed an increased electron transport efficiency (ETo/ABS) and the probability of the contribution of electrons transported to captured electrons (ETo/TRo). Meanwhile, the Cu treatment reduced the density of reactive centers (RC/ABS).

### 2.4. The Effect of Metal Treatments on Biochemical Parameters

#### 2.4.1. Rhizosphere

The total phenolic (TP) content in the rhizosphere of control plants was significantly higher than for other variants, while for Al treatments, TP content was the lowest (Table 3). The individual phenolic compounds were below the detection limit.

The main LMWOAs were citric and succinic (Table 3). The Pb, Zn, and Ni treatments led to a significant decrease in citric acid content compared to the control, especially in the case of Ni treatment. Conversely, Al treatment caused a significant increase in citric acid content. Regarding succinic acid, Cu treatment increased its content in the rhizosphere, while Ni treatment decreased it.

#### 2.4.2. Roots

The TP content in roots was comparable in control, Zn-, and Ni-treated plants (Table 4). A significant increase was noted for other metals, with the highest value for Pb treatment. The phenolic profile of control roots comprised only *p*-coumaric acid and catechin. Metal treatments induced changes in the phenolic profile, leading to the synthesis of new compounds. Rutin, *p*-coumaric, and syringic acids were detected in the roots of Pb-treated plants, while catechin and *p*-coumaric acid were quantified under Zn treatment. The most different pattern of phenolic composition in roots was confirmed for Cu-treated plants with gallic and *p*-coumaric acids (as dominant), vanillic, syringic, and ferulic acids. The phenolic profile of Al-treated plants included only *p*-coumaric acid with content similar to the control and catechin content lower than the control. In the phenolic profile of the roots of plants treated with Ni, only *p*-coumaric acid was identified.

Among LMWOAs, citric and succinic acids were dominant, and malonic, lactic, and malic acids were also found in roots (Table 4). Compared to the control, a significant decrease in citric and succinic acid content was noted for Pb, Zn, Cu, Al, and Ni treatments. The Cu treatment resulted in a significant increase in the content of malonic acid compared to the control plants. In contrast, Zn treatment stimulated the synthesis of lactic acid, while Ni stimulated malic acid synthesis.

#### 2.4.3. Shoots

The metal treatments significantly increased the TP content in shoots, with the highest value for Ni treatment (Table 5). The dominant phenolic compound in control was 2,5-DHBA, followed by rutin, quercetin, 4-HBA, ferulic acid, *t*-cinnamic acid, *p*-coumaric acid, chlorogenic acid, protocatechuic acid, catechin, syringic acid, kaempferol, sinapic, vanillic, caffeic, and gallic acids. The Pb treatment resulted in a drop in the content of most phenolic compounds, except for enhanced synthesis of syringic acid. The dominant compounds of Pb-treated plants were *t*-cinnamic acid, syringic acid, 2,5-DHBA, and rutin. For Zn treatment, rutin was the main phenolic compound. Significant elevation of protocatechuic and syringic acids and kaempferol, along with the reduction of gallic, 4-HBA, sinapic, 2,5-DHBA, chlorogenic, *p*-coumaric, ferulic, sinapic acids, and catechin content compared to control was observed. The phenolics profile for Cu, Al, and Ni treatments contained rutin as a dominant compound. The content of protocatechuic, syringic, *t*-cinnamic, and caffeic acids, rutin, and kaempferol in Cu-treated plants significantly increased compared to the control. The phenolic profile of Al-treated plants included significantly higher contents of protocatechuic, vanillic, syringic, *t*-cinnamic, and caffeic acids, rutin, catechin, and kaempferol compared to the control. The enhanced synthesis of most phenolic compounds (protocatechuic, vanillic, syringic, *t*-cinnamic, chlorogenic, caffeic and *p*-coumaric acids, catechin, rutin, quercetin, and kaempferol) was confirmed for Ni-treated plants.

Salicylic acid was detected in shoots ranging from 0.27 to 1.21 µg g^−1^ FW, depending on the treatment (Table 5). The highest level of the metabolite was determined for Cu treatment and was ~4 higher than for control plants. A significant increase in SA content was also noted for Pb- and Zn-treated plants, while the SA level was comparable to the control for Al and Ni treatments.

The dominant LMWOAs in the control shoots were succinic, citric, and malonic (Table 5). Their content significantly decreased for Pb, Zn, and Al treatments compared to the control. The lowest content of malonic acid was found in Al-, citric acid in Pb-, and succinic acid in Zn-treated plants. The content of the primary LMWOAs significantly differed from the control in Cu and Ni treatments. A significant increase in citric acid content was observed for the Cu treatment. Meanwhile, Ni treatment led to a significant rise in malonic acid content. Additionally, malic acid was not detected in the control, while the metal addition significantly increased its content in shoots. The lowest malic acid content among metal-treated plants was noted for the Pb, while the highest was for Cu treatment. Oxalic, lactic, acetic, and fumaric acids were detected at much lower concentrations.

The fructose and sucrose content significantly increased in the shoots of metal-treated plants (Table 5). The significant elevation of glucose content was noted for Cu treatment, while the drop was confirmed for Zn and Al treatments.

### 2.5. PCA Analysis

**Figure 2:**
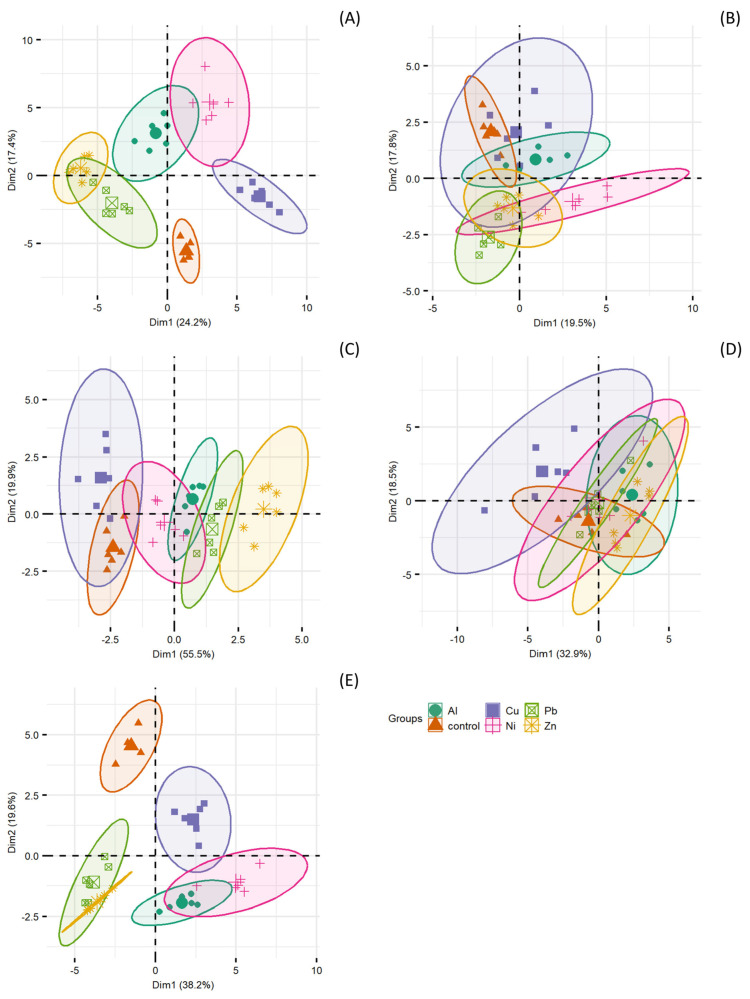
Separated groups based on PCA analysis for (**A**) all parameters, (**B**) minerals, (**C**) biomass, (**D**) chlorophyll fluorescence and gas exchange, (**E**) sugars, low−molecular−weight organic acids (LMWOAs), and phenolic compounds.

Based on the groups separated in the PCA analysis covering all parameters (Figure 2A), it was demonstrated that the metal treatments induced variations in the tested parameters. The groups for metals differed significantly from the control group. The plant’s response under Pb and Zn treatments exhibited partial similarity. In contrast, the Cu, Ni, or Al treatments caused a reaction specific to each metal. The separated groups in the PCA analysis for shoot mineral content (Figure 2B), biomass (Figure 2C), chlorophyll fluorescence and gas exchange (Figure 2D) were less specific and more similar to the control group, especially for chlorophyll fluorescence and gas exchange data. The separated control group had a common part with all groups separated for metals. Regarding biomass parameters, the highest similarity to the control was shown for Cu, lower for Ni, and other metals; the separated groups differed significantly from the control. The analysis of the groups separated for sugars, organic acids, and phenolic compounds (Figure 2E) indicated a largely specific plant response compared to the control, with the separated groups of Pb and Zn and Al and Ni being similar, as noted in the large common part of these groups. The biplots showing particular variables employed for the PCA analysis and their contribution to the variance explained by the first and second axes were presented in Supplementary Materials Figure S1.

## 3. Discussion

*P. nigra* L. × *P. maximowiczii* plants exposed to specific doses of a single metal in soil (equimolar concentrations of Pb, Zn, Al, Ni, and Cu, i.e., 500 mL of 4 *mM* solutions of single metal salts: (Pb(NO_3_)_2_; Zn(NO_3_)_2_ × 6H_2_O; Al(NO_3_)_3_ × 9H_2_O; Ni(NO_3_)_2_ × 6H_2_O; or Cu(NO_3_)_2_ × 3H_2_O) for four weeks showed no growth inhibition, with an increase in biomass compared to the control. Cu treatments exhibited the highest ratio of shoot and root biomass, while Ni treatments showed the lowest ratio, indicating variations in the proportion of these organs in the overall biomass increase. The stimulation of plant growth resembled the hormesis effect, where sublethal concentrations of toxic metals acted as an adaptive mechanism, helping the plants cope with stress and promoting their growth [29,30].

The addition of specific metals to the soil led to increased metal content in roots, rods, and shoots of *P. nigra* L. × *P. maximowiczii*, primarily accumulating in roots. Migeon et al. (2012) [26] revealed the second-highest tolerance of *P. nigra* L. × *P. maximowiczii* in a screening hydroponic study on 21 poplar hybrids to Cd, Zn, Cu, or Ni. A moderate metal uptake accompanied the high tolerance of the hybrid compared to the other genotypes. Zn was accumulated to the greatest extent among investigated metals, followed by Ni > Cu > Cd. In the field study, Kacálková et al. (2015) [31] also reported the highest uptake of Zn to leaves of *P. nigra* L. × *P. maximowiczii* compared to Cu and Cd, and similar was observed in our study. In another study [32], in contrast to Zn, Ni uptake was greatly restricted to roots in plants as recorded from field investigations.

Photosynthesis and gas exchange are very sensitive to the toxicity of metals, and the decreases of the processes and related parameters were observed [33]. In *P. nigra* L. × *P. maximowiczii*, applied doses of metal salt induced minimal changes in physiological parameters such as photosynthesis, gas exchange, fluorescence, and associated parameters. Only exposure to Pb decreased net photosynthesis. The significant reduction in stomatal conductance occurred solely with Zn treatment, whereas a decrease in transpiration was noted with Cu treatment. Metal stress negatively impacts photosynthesis by altering chloroplast structure, inhibition of the Calvin cycle, stomatal closure, leading to CO_2_ deficiency, disruptions in nutrient uptake, and hindrance in chlorophyll synthesis [33]. The enhancement of photosynthesis rates observed in the Zn, Cu, and Al treatments contradicted findings reported in existing studies [34,35]. The observed effect was similar to hormesis. Some authors argue that there is a transient enhancement of gas exchange at low doses of the stressor [36,37]. The intensified gas exchange at low doses of stressors may result from improved nutrient absorption, supporting the supply of nutrients to aboveground tissues and countering adverse environmental conditions [37,38]. For the Pb and Ni treatments, the transition from stimulation to growth inhibition with increasing metal content in the tissues was shorter than for Zn, Cu, and Al treatments. The results suggest that a longer experiment might also have slowed down the growth of Zn-, Cu-, and Al-treated plants, confirming previous observations on the different effects of these metals on fluorescence kinetics [39]. Metals exhibited no negative impact on the OJIP test parameters (TRo/ABS, Fv/Fo, PIABS, Sm), although some authors have noted that PIABS is sensitive to Cu and Zn [40,41]. Singh et al. (2022) [42] observed a decrease in Fv/Fo, quantum yield for electron transport, and quantum yield of primary photochemistry, but an increase in the dissipated quantum yield with higher Cu concentrations. Cu treatment in *P. nigra* L. × *P. maximowiczii* decreased RC/ABS and increased ETo/ABS and ETo/TRo, while Zn treatment increased RC/ABS and DIo/RC. Studies showed a dose-response relationship for Cu stress on ABS/CSM, TRO/CSM, ETO/CSM, and maximum quantum yield, with performance indices rising at lower Cu concentrations but decreasing at higher ones [42]. Chen et al. (2022) [40] found a significant decrease in TRo/ABS under Cu stress, unaffected by Zn. In Cu- and Zn-stressed plants, reduced RC/CSM increased ABS/RC and TRo/RC, and elevated DIo/RC indicated a self-protection mechanism that eliminates excess energy in PSII and enhances heat dissipation. Pb and Al treatments had no impact on the OJIP test parameters of *P. nigra* L. × *P. maximowiczii*. This does not confirm earlier reports of a strong response of these parameters to sublethal doses of Pb [43] or Al [44]. Our study demonstrated an increase in ETo/ABS and ETo/TRo after applying Ni, which is inconsistent with previous reports [45].

Accumulation of phenolic compounds in plant tissues and their exudation into the rhizosphere is observed under various environmental stresses, including exposure to metals [46,47,48,49]. The exudation of phenolic compounds into the rhizosphere by *P. nigra* L. × *P. maximowiczii* was low and inhibited by metal addition. In the rhizosphere, the role of phenolic compounds is significant, as they can participate in defense mechanisms due to their antioxidant properties. Their ability to chelate metals eliminates toxic ions during metal stress and also improves soil properties due to their contribution to the mineralization and humification of the soil by increasing the mobility and bioavailability of nutrients, including iron, molybdenum, and Cu [50,51,52]. The TP content increased in the roots for Pb, Cu, and Al treatments. The most notable changes in TP content and greater variation in phenolic compound composition under metal treatments were observed in the shoots, with the profile revealing hydroxybenzoic acids, hydroxycinnamic acids, and flavonoids in both roots and shoots. The increase in content and synthesis of novel phenolic compounds in plants exposed to metal, compared to the control, can be linked to the role of these metabolites in response to stress. During metal stress, phenolic compounds can function as antioxidants, demonstrating the capability to scavenge free radicals, chelate metal ions, and donate electrons or hydrogen atoms. The effectiveness of these actions depends on the structural features, specifically the number and position of the hydroxyl and carboxyl groups, with hydroxycinnamic acids (C6–C3 structure) exhibiting greater antioxidant ability than hydroxybenzoic acids [53,54]. Gallic acid is a potent antioxidant. However, its content decreased in shoots following metal treatments. A significant increase in protocatechuic acid (C6–C1 structure) in shoots of metal-treated plants resulted from its chelating and antioxidant properties, likely aimed at mitigating the effects of metal-induced oxidative stress [55,56]. Caffeic, cinnamic, and vanillic acids contribute to the antioxidant system of plants, and their accumulation was observed under Cu, Al, and Ni treatments. The increase in phenolic compound content indicates their involvement in the activation of defense mechanisms and the enhanced activation of the phenylpropanoid synthesis pathway by these metals [48,57]. Salicylic acid belongs to phenolic metabolites and serves a phytohormone-like function in regular plant growth and development. Under biotic and abiotic stress conditions, an intense metabolite biosynthesis occurs to regulate plant response to the stressor and enable survival [58,59]. Our previous studies examined the impact of Cu and Ni on energy willow in a hydroponic culture. Both metals triggered the accumulation of salicylic acid in leaves, with levels increasing with the metal concentrations in the medium, reaching ~8 and 24 µg g^−1^ FW for Cu and Ni, respectively [60,61]. This was accompanied by a severe deterioration in plant growth and the manifestation of symptoms indicative of metal toxicity. Furthermore, the impact of Cu was markedly influenced by the Ca:Mg ratio [62]. In the present study, the basal content of the metabolite was comparable to that of the control plants of willow. In contrast, relatively lower accumulation was noted for metal-treated plants than for willow and was accompanied by reduced metal toxicity. The results suggest a high tolerance of the investigated poplar to metals, with Cu inducing the strongest plant reaction.

LMWOAs are essential in the transport, storage, and tolerance of metals such as Al, Cd, Cr, Cu, Pb, Ni, and Zn [63,64]. Their presence and specific profile in the rhizosphere may represent one of the initial responses of plants to metal toxicity [65]. In a study on the rhizosphere of *P. nigra* L. × *P. maximowiczii*, the LMWOAs profile depended on the type of metal introduced into the soil, with citric and succinic acids being the predominant acids. The exuded acids exhibit a strong affinity for metals, leading to the formation of complexes in the rhizosphere and facilitating metal stress alleviation and metal ion sequestration [66,67,68,69,70]. Furthermore, LMWOAs play a key role in the transport of metals within root tissues, facilitating their translocation to the xylem and aiding in vacuolar sequestration [71]. In the roots of *P. nigra* L. × *P. maximowiczii*, five LMWOAs were identified, with citric and succinic acids as the dominant ones, whose concentrations significantly decreased under Pb, Zn, Cu, and Al treatment, confirming their ability to form stable complexes with metal ions in plant tissues [72].

Higher LMWOAs content was observed in the shoots than in the rhizosphere and roots, with citric, succinic, and malonic acids as the main components. These acids are important intermediates in the tricarboxylic acid (TCA) cycle, and their increased production in leaves is part of biochemical adjustments aimed at mitigating metal toxicity [73]. Elevated LMWOAs concentrations in plant leaves are associated with biomass reduction and/or increased metal accumulation, as confirmed by our research and supported by literature reports on plants such as *Salix*, *Pinus sylvestris*, *Sesuvium portulacastrum*, *Brassica juncea*, and Bermuda grass [74,75]. Changes in LMWOAs production in response to increased Cu, Pb, or Zn accumulation suggest their role in metal detoxification mechanisms in the rhizosphere, root tissues, and subsequently in aerial organs, depending on the specific metal [69,76,77,78].

The results for *P. nigra* L. × *P. maximowiczii* show the accumulation of fructose, sucrose, and glucose in leaves under metal stress (particularly under Cu treatment), confirming previous studies [12,79]. Sugars play an essential role in defending against oxidative stress by protecting plant cells from damage and helping maintain osmotic pressure, crucial for cell integrity [80,81,82,83]. Additionally, as an energy source, they support repair and defense processes, and their accumulation triggers signaling responses associated with the plant’s defensive mechanisms [84,85,86,87,88].

## 4. Materials and Methods

### 4.1. Plant Material

A Japanese poplar hybrid (*Populus nigra* L. × *P. maximowiczii*) was obtained from the AgroWOOD company (Janikowo, Poland). Stem cuttings (20 cm long) from non-branching annuals were used for plant vegetative propagation, prepared according to the European Commission standard (EC 2000) [89], and were at least 1 cm thick at the thinner end.

### 4.2. Pot Experiment

The cuttings were rooted for 14 days in Knop’s solution (half strength) [90] and then transferred to the standard garden soil (pH 5.5–6.5; EC < 1.5 mS/m; fraction 0–20) (Bioprodukty sp. z o.o., Parsęcko, Poland) in plastic pots. The cuttings were treated with 500 mL of tap water (control) or 500 mL of 4 mM solutions of single metal salts: (Pb(NO_3_)_2_; Zn(NO_3_)_2_ × 6H_2_O; Cu(NO_3_)_2_ × 3H_2_O; Al(NO_3_)_3_ × 9H_2_O; or Ni(NO_3_)_2_ × 6H_2_O). Based on the dry matter content and soil moisture weight per pot, the following metal concentrations were obtained: 323.75 (Pb), 102.21 (Zn), 99.29 (Cu), 91.71 (Ni), and 42.16 (Al) mg kg^−1^ DW. The resultant values were higher than the majority of recorded samples in a 25-year study of 216 sampling points in Poland [91].

The greenhouse experiment was conducted under controlled conditions with 6 replicates organized into 2 blocks. After four weeks, plants were harvested and separated into shoots, rods, and roots. Fresh biomass parameters, shoot number, and length were then measured.

### 4.3. Gases and Reagents

High-purity argon was employed as a plasma gas. All reagents for determination of elements were diluted with high-purity deionized water obtained from the Milli-Q water purification system (Merck Millipore Darmstadt, Germany). ICP commercial analytical standards (Romil, Cambridge, UK) were used for calibration. The 65% nitric acid for mineralization of samples was purchased from Merck, Germany. The standards of phenolic compounds (gallic acid ≥98%, protocatechuic acid ≥ 98.99%, vanillic acid ≥ 97%, 4-HBA ≥ 99%, 2,5–DHBA ≥98%, syringic acid ≥ 98%, catechin ≥ 98%, p-coumaric acid ≥ 98%, ferulic acid ≥ 98%, chlorogenic acid ≥ 95%, sinapic acid ≥ 97%, trans-cinnamic acid ≥ 99%), and organic acids (acetic ≥ 99.7%, fumaric ≥ 99%, citric ≥ 99.5–100.5%, malic ≥ 99%, malonic (certified reference material TraceCERT^®^), lactic ≥ 85%, oxalic ≥ 99%, quinic (analytical standard) and succinic ≥ 99.5%) and sugars (sucrose ≥ 99.5%, fructose ≥ 99%, glucose ≥ 99.5%) were purchased from Sigma–Aldrich (Steinheim, Germany). Caffeic acid (certified reference material TraceCERT^®^) was obtained from Sigma–Aldrich (Basel, Switzerland). All salts used for the analysis, including lead nitrate (Pb(NO_3_)_2_), zinc nitrate hexahydrate (Zn(NO_3_)_2_ × 6H_2_O), copper nitrate trihydrate (Cu(NO_3_)_2_ × 3H_2_O), aluminum nitrate nonahydrate (Al(NO_3_)_3_ × 9H_2_O), and nickel (II) nitrate hexahydrate (Ni(NO_3_)_2_ × 6H_2_O), were of analytical reagent grade and obtained from Chempur (Piekary Śląskie, Poland). Hydrochloric acid (HCl, 35–38%, analytical reagent grade) was obtained from Poch (Bydgoszcz, Poland).

### 4.4. Determination of Elements

#### 4.4.1. Sample Preparation

The following procedure was conducted according to Proch et al. (2023) [92]. Samples of shoots, rods, and roots were weighed (0.300 ± 0.001 g) and quantitatively transferred to Teflon containers (55 mL). Subsequently, 7.0 mL of 65% nitric acid was added. The samples underwent digestion using the Mars 6 Xpress microwave digestion system (CEM, Atthews, NC, USA) in three stages: (1) ramping the temperature (20 min), (2) holding at 180 °C (20 min), and (3) cooling (20 min). Following digestion, the samples were diluted to achieve a final volume of 15 mL.

#### 4.4.2. Instrumentation

An inductively coupled plasma optical emission spectrometer, Agilent 5110 ICP–OES (Agilent, Santa Clara, CA, USA), was used to analyze the samples. The standard conditions were used in all measurements: radio frequency (RF) power was 1200 W, and gas (argon) flow rates were 12 L min^−1^, 0.7 L min^−1^, and 1.0 L min^−1^ for plasma, nebulizer, and auxiliary, respectively. Echelle grading fixed optic was thermostated at 35 °C, and a detector, VistaChip II with the Charge Coupled Device (CCD), was cooled to −40 °C using a triple Peltier system. The signal was measured in 3 replicates, and the accusation time was 5 s. The plasma torch view was the synchronous vertical dual view (SVDV), which allowed for the simultaneous axial and radial view using dichroic spectral combiner (DSC) technology. The viewing height view observation was 8 mm for the radial view. The determination was provided at the following emission lines: Al 396.152 nm, Ca 422.673 nm, Cu 327.395 nm, Fe 238.204 nm, K 766.491 nm, Mg 279.553 nm, Na 588.995 nm, Ni 231.604 nm, Pb 220.353 nm, and Zn 213.857 nm. Detection limits (DLs) were estimated in the range from 0.002 to 2.689 mg kg^−1^ dry weight (DW) using 3-sigma criteria: Al 0.171; Ca 2.689; Cu 0.009; Fe 0.039; K 2.051; Mg 0.513; Na 0.331; Ni 0.002; Pb 0.101; and Zn 0.196 mg kg^−1^. The uncertainty for the complete analytical process (including sample preparation) was 20%. Certified reference materials (CRMs) representing wood (NIST SRM 2790, Gaithersburg, MD, USA) and leaves (INCT-TL-1, Warsaw, Poland) were used in quality control with acceptable recovery (80–120%) for most of the determined elements (Supplementary Materials Table S1).

### 4.5. Physiological Parameters

Gas exchange and chlorophyll fluorescence measurements were performed 1–2 days before the end of the experiment on young, fully expanded leaves. Leaf gas exchange was measured using a portable infrared gas analyzer coupled to a broad chamber (LCpro+, ADC, Hoddesdon, UK). The chamber leaf area was 6.25 cm^2^ with a flow rate of 200 mL min^−1^. The net photosynthetic rate (A), transpiration (E), stomatal conductance (g_s_), and internal CO_2_ concentration (C_i_) were automatically calculated by the LCpro+. Measurements were made at 25 °C, 380 ppm CO_2_, PPFD 1000 μmol m^−2^ s^−1^, and ambient air humidity. Chlorophyll fluorescence was measured after a 20-min dark adaptation using detachable leaf clips [93]. The OJIP test was performed with an OS5p fluorometer (OptiSciences Inc., Hudson, NY, USA), and the light curves (LC) with a FluorPen FP 110/D fluorometer (Photon System Instruments, Drásov, Czech Republic). The OJIP test is a method for analyzing chlorophyll fluorescence in plants under abiotic stresses. It helps assess plant physiological state and detect unfavorable environmental conditions such as light, temperature, drought, soil salinity, mineral deficiency, or metal toxicity. The OJIP points on the induction curve, at which the fluorescence signal is measured, are as follows: O (origin of the fluorescence transient), J (intermediate inflection point), I (peak of the fluorescence transient), and P (plateau level of fluorescence).

### 4.6. Determination of Phenolic Compounds, Low-Molecular-Weight Organic Acids (LMWOAs), and Soluble Sugars

#### 4.6.1. Extraction from Rhizosphere, Roots, and Shoots

Rhizosphere samples were obtained by gently shaking the root systems and carefully scraping the soil from the roots to collect the soil that closely adhered to the roots. To 50 g of a dry soil sample, acidified water (pH 2.0) was added and shaken (at room temperature for 12 h). Water extracts were filtered (Whatman No. 42 filters, Global Life Sciences Solutions USA LLC, Wilmington, NC, USA) and extracted three times with ethyl acetate (20 mL, 5 min) [94]. The organic solvent volume was reduced to 5 mL using an evaporator, then transferred to an amber glass vial and evaporated to dryness at room temperature. Before chromatographic analysis, the dried samples of rhizosphere were redissolved in 1 mL of deionized water.

The powdered (homogenized in liquid nitrogen) samples of roots or shoots (leaves and stems) were mixed with 80% methanol with 36% hydrochloric acid (99:1, *v*/*v*). The mixtures were sonicated for 30 min at 40 °C, shaken for 7 h using an orbital shaker, and then centrifuged at 3600× *g* for 15 min at room temperature. The dried samples were subsequently redissolved in 80% methanol before analysis.

#### 4.6.2. Chromatographic Analyses

Phenolic compounds and LMWOAs were analyzed using a Waters Acquity H class UPLC system coupled with a Waters Photodiode Array Detector (Waters Corporation, Milford, MA, USA). The separation of metabolites was accomplished by employing a Waters Acquity UPLC BEH C18 column (150 × 2.1 mm, 1.7 μm) at λ = 280 and 320 nm, utilizing a gradient elution with water and acetonitrile (both containing 0.1% formic acid) at a flow rate of 0.4 mL min^−1^ [18]. Detection was performed using an external standard on a Waters Photodiode Array Detector (Waters Corporation, Milford, MA, USA) at λ = 280 nm (gallic acid, protocatechuic acid 4-HBA, *t*-cinnamic acid, syringic acid, vanillic acid, catechin, and LMWOAs) and λ = 320 nm (ferulic acid, caffeic acid, *p*-coumaric acid, chlorogenic acid, sinapic acid). For salicylic acid determination, a Waters Alliance 2695 Chromatograph coupled with a Waters 2475 Multi-λ Fluorescence Detector (Waters Corporation, Milford, MA, USA) and a Spherisorb ODS2 column (100 × 4.6 mm, 5 μm) were used with potassium acetate buffer (pH 5.0) as a mobile phase [20]. Peak identification was carried out by comparing retention times with those of chemical standards. For UPLC analyses, the detection limits (DL) were calculated based on a signal-to-noise ratio of 3:1. Raw data were collected and analyzed using Empower 3 software [95].

#### 4.6.3. Total Phenolic Assay

The methanolic extracts were combined with 1 mL of Folin–Ciocalteu phenol reagent (diluted with H_2_O; 1:1, *v*/*v*). After 3 min, 1 mL of 20% Na_2_CO_3_ was introduced. The mixture was incubated for 30 min in darkness at room temperature. Subsequently, the absorbance of the samples was measured at λ = 765 nm using a Cary 300 Bio UV-Vis scanning spectrophotometer (Agilent Technologies, Santa Clara, CA, USA) [95].

#### 4.6.4. Sugars Determination

The contents of glucose, fructose, and sucrose were determined in methanolic extracts using a Waters Alliance 2695 system coupled with a Waters 2414 Refractive Detector (Waters Corporation, Milford, MA, USA). Sugar separation was accomplished using a SupelcosilTM LC-NH2 Column (250 × 4.6 mm, 5 μm). A mobile phase consisting of water and acetonitrile (25:75%, *v*/*v*) was employed at a flow rate of 1.0 mL min^−1^. The identification of peaks was carried out by comparing the retention times with those of glucose, fructose, and sucrose standards [96].

### 4.7. Statistics

The significance of the effect of treatments on the analyzed parameters was assessed using an ANOVA analysis. Differences between mean values were estimated using a Newman–Keuls post hoc test at a significance level of *p* = 0.05. The analysis was performed for a single-factor experiment in a randomized block design in 6 replicates. Principal component analysis (PCA) was conducted in the R environment (RStudio 2023.09.1+494 by Posit Software, PBC and R software (ver. 4.3.2) by the R Foundation for Statistical Computing) using the FactoMineR package ver 2.6 [97], and the factoextra package ver. 1.0.7 was used to extract and visualize the results.

## 5. Conclusions

In conclusion, the stimulatory effect of metal treatments in *P. nigra* L. × *P. maximowiczii* was observed, which was evident in the measurements of plant biomass and photosynthetic rate. The results of the OJIP test indicated no impact of Pb or Al and a slightly positive effect of Zn, Cu, or Ni on PSII. This was reflected by a relatively weak induction of salicylic acid biosynthesis in shoots and observed only for selected metals (Cu > Pb~Zn). The content of other analyzed bioactive compounds, including phenolic compounds, low-molecular-weight organic acids, and sugars, exhibited numerous changes depending on the metal employed. The findings suggest the potential for utilizing *P. nigra* L. × *P. maximowiczii* in dendroremediation efforts aimed at soils contaminated with the metals under analysis at moderate levels. Further research and field studies would be needed to more comprehensively assess the efficacy and limitations of this approach in real-world soil remediation scenarios.

## Figures and Tables

**Table 1:** Content of elements in *P. nigra* L. × *P. maximowiczii* hybrid biomass after 4 weeks of growth in soil enriched with selected metal salts.

Element [mg kg^−1^ DW]	Control	Treatment				
		Pb	Zn	Cu	Al	Ni
	Roots					
Pb	5.71 ^b^ ± 0.30	109.8 ^a^ ± 11.59	2.61 ^b^ ± 0.03	3.99 ^b^ ± 0.58	3.54 ^b^ ± 0.21	2.81 ^b^ ± 0.08
Zn	50.08 ^bc^ ± 2.46	47.53 ^bc^ ± 3.28	83.11 ^a^ ± 4.11	44.08 ^bc^ ± 1.91	54.15 ^b^ ± 5.90	39.24 ^c^ ± 1.24
Cu	11.79 ^b^ ± 0.38	10.66 ^b^ ± 0.53	10.90 ^b^ ± 0.49	73.78 ^a^ ± 2.22	9.84 ^b^ ± 0.58	8.45 ^b^ ± 0.66
Al	612.2 ^c^ ± 32.10	566.1 ^c^ ± 16.20	537.8 ^c^ ± 53.00	886.6 ^b^ ± 16.90	1301 ^a^ ± 42.60	539.9 ^c^ ± 20.80
Ni	1.92 ^b^ ± 0.10	1.14 ^c^ ± 0.05	1.09 ^c^ ± 0.08	1.50 ^bc^ ± 0.06	1.32 ^c^ ± 0.06	17.56 ^a^ ± 0.49
	rods					
Pb	bdl	0.88 ^a^ ± 0.14	bdl	0.31 ^b^ ± 0.06	bdl	bdl
Zn	42.55 ^bc^ ± 1.92	47.13 ^b^ ± 3.98	58.05 ^a^ ± 7.77	38.17 ^c^ ± 4.57	49.48 ^b^ ± 5.10	47.41 ^b^ ± 5.54
Cu	2.99 ^b^ ± 0.33	2.17 ^c^ ± 0.09	3.12 ^b^ ± 0.40	3.811 ^a^ ± 0.50	2.41 ^c^ ± 0.36	2.45 ^c^ ± 0.33
Al	24.69 ^a^ ± 1.79	22.75 ^a^ ± 1.81	23.94 ^a^ ± 2.85	26.13 ^a^ ± 3.59	26.94 ^a^ ± 3.48	25.00 ^a^ ± 1.12
Ni	0.14 ^b^ ± 0.03	0.18 ^b^ ± 0.02	0.20 ^b^ ± 0.04	0.17 ^b^ ± 0.04	0.14 ^b^ ± 0.02	1.21 ^a^ ± 0.23
	shoots					
Pb	bdl	0.49 ^a^ ± 0.08	bdl	bdl	bdl	bdl
Zn	78.42 ^b^ ± 9.03	63.55 ^b^ ± 3.33	140.65 ^a^ ± 13.96	68.03 ^b^ ± 13.06	74.22 ^b^ ± 11.69	76.18 ^b^ ± 9.75
Cu	4.28 ^b^ ± 0.88	3.24 ^c^ ± 0.50	3.20 ^c^ ± 0.41	5.34 ^a^ ± 0.61	3.08 ^c^ ± 0.80	3.12 ^c^ ± 0.41
Al	36.53 ^b^ ± 1.51	32.74 ^b^ ± 3.17	32.98 ^b^ ± 2.97	41.91 ^b^ ± 6.10	61.99 ^a^ ± 9.77	33.47 ^b^ ± 2.88
Ni	0.37 ^b^ ± 0.06	0.23 ^b^ ± 0.05	0.24 ^b^ ± 0.08	0.46 ^b^ ± 0.16	0.23 ^b^ ± 0.01	2.14 ^ab^ ± 0.68

n = 3; identical superscripts (a, b, c) denote non-significant differences between means in rows according to the post hoc Newman–Keuls test; bdl—below detection limit.

**Table 2:** Gas exchange parameters, light curves based on chlorophyll fluorescence and chlorophyll fluorescence measurements for the OJIP test of *P. nigra* L. *× P. maximowiczii* hybrid biomass after 4 weeks of growth in soil enriched with selected metal salts.

Parameter	Control	Treatment				
		Pb	Zn	Cu	Al	Ni
A [μmol m^−2^ s^−1^]	13.48 ^b^ ± 0.66	11.98 ^c^ ± 0.98	15.45 ^a^ ± 0.86	15.53 ^a^ ± 1.23	14.58 ^a^ ± 0.80	12.73 ^bc^ ± 0.70
g_s_ [mol m^−2^ s^−1^]	0.28 ^ab^ ± 0.07	0.29 ^a^ ± 0.05	0.21 ^c^ ± 0.02	0.23 ^bc^ ± 0.05	0.26 ^a–c^ ± 0.04	0.247 ^a–c^ ± 0.05
E [mmol m^−2^ s^−1^]	2.49 ^a^ ± 0.20	2.31 ^ab^ ± 0.17	2.34 ^ab^ ± 0.12	2.13 ^b^ ± 0.18	2.33 ^ab^ ± 0.27	2.288 ^ab^ ± 0.21
C_i_ [vpm]	230.4 ^a^ ± 20.60	235.9 ^a^ ± 9.20	217.5 ^a^ ± 20.18	216.7 ^a^ ± 11.00	231.6 ^a^ ± 17.60	231.2 ^a^ ± 13.40
ETR_PAR100 [μmol m^−2^ s^−1^]	18.95 ^a^ ± 1.09	17.85 ^ab^ ± 1.39	16.92 ^ab^ ± 0.79	18.28 ^ab^ ± 1.79	16.22 ^b^ ± 1.14	17.52 ^ab^ ± 0.95
ETR_PAR200 [μmol m^−2^ s^−1^]	26.51 ^ab^ ± 7.73	26.25 ^ab^ ± 1.90	24.79 ^bc^ ± 1.39	28.35 ^a^ ± 2.32	23.35 ^c^ ± 1.45	24.80 ^bc^ ± 1.16
ETR_PAR300 [μmol m^−2^ s^−1^]	35.46 ^bc^ ± 2.84	37.53 ^b^ ± 1.30	33.79 ^cd^ ± 1.16	42.06 ^a^ ± 1.95	32.54 ^d^ ± 2.17	37.92 ^b^ ± 2.30
ETR_PAR50 [μmol m^−2^ s^−1^]	52.12 ^b^ ± 5.07	52.03 ^b^ ± 1.78	45.87 ^c^ ± 2.10	63.22 ^a^ ± 4.14	46.71 ^c^ ± 2.15	50.21 ^bc^ ± 2.57
ETR max1000 [μmol m^−2^ s^−1^]	60.33 ^b^ ± 5.79	63.50 ^b^ ± 3.30	54.00 ^c^ ± 3.51	81.50 ^a^ ± 5.65	62.58 ^b^ ± 2.78	63.83 ^b^ ± 2.27
Fv/Fo	4.936 ^a^ ± 0.12	4.94 ^a^ ± 0.29	4.75 ^a^ ± 0.43	4.65 ^a^ ± 0.29	5.01 ^a^ ± 0.32	4.63 ^a^ ± 0.29
Mo or RC/ABS	0.69 ^b^ ± 0.07	0.70 ^b^ ± 0.04	0.82 ^a^ ± 0.04	0.53 ^c^ ± 0.07	0.76 ^ab^ ± 0.06	0.68 ^b^ ± 0.05
ABS/RC	1.70 ^ab^ ± 0.21	1.84 ^ab^ ± 0.18	2.07 ^a^ ± 0.11	1.58 ^b^ ± 0.23	1.99 ^a^ ± 0.21	1.93 ^ab^ ± 0.22
TRo/RC	1.41 ^ab^ ± 0.17	1.53 ^ab^ ± 0.15	1.71 ^a^ ± 0.10	1.30 ^b^ ± 0.20	1.66 ^a^ ± 0.17	1.58 ^ab^ ± 0.19
ETo/RC	0.72 ^a^ ± 0.11	0.83 ^a^ ± 0.12	0.89 ^a^ ± 0.08	0.77 ^a^ ± 0.14	0.90 ^a^ ± 0.12	0.91 ^a^ ± 0.16
DIo/RC	0.29 ^b^ ± 0.04	0.31 ^ab^ ± 0.03	0.36 ^a^ ± 0.03	0.28 ^b^ ± 0.03	0.33 ^ab^ ± 0.05	0.34 ^ab^ ± 0.30
TRo/ABS	0.83 ^a^ ± 0.01	0.83 ^a^ ± 0.01	0.82 ^a^ ± 0.01	0.82 ^a^ ± 0.01	0.83 ^a^ ± 0.01	0.82 ^a^ ± 0.01
ETo/ABS	0.43 ^c^ ± 0.02	0.45 ^a–c^ ± 0.02	0.43 ^bc^ ± 0.02	0.48 ^a^ ± 0.03	0.45 ^a–c^ ± 0.02	0.47 ^ab^ ± 0.03
DIo/ABS	0.17 ^a^ ± 0.01	0.17 ^a^ ± 0.01	0.18 ^a^ ± 0.01	0.18 ^a^ ± 0.01	0.17 ^a^ ± 0.01	0.18 ^a^ ± 0.01
ETo/TRo	0.51 ^c^ ± 0.02	0.54 ^bc^ ± 0.02	0.52 ^c^ ± 0.02	0.59 ^a^ ± 0.03	0.54 ^bc^ ± 0.02	0.57 ^ab^ ± 0.03

n = 6; identical superscripts (a, b, c) denote non-significant differences between means in rows according to the post hoc Newman–Keuls test; A—photosynthetic rate; g_s_—stomal conductance; E—transpiration; C_i_—internal CO_2_ concentration; TRo/ABS—maximal specific trapping flux; Fv/Fo—maximum water splitting efficiency on the donor side of the PSII; PAR—photosynthetic active radiation; ETR_PAR—electron transport rate; ETR max1000—maximum ETR; Mo or RC/ABS—density of reaction centers per absorbed photons; ABS/RC—absorption photon flux per reaction center; TRo/RC—trapping flux in PS II (dark adapted sample) per reaction center; ETo/RC—electron transport beyond QB (dark-adapted sample) per reaction center; DIo/RC—dissipation from PS II (dark adapted sample) per reaction center; TRo/ABS—maximum quantum yield of primary PSII photochemistry; ETo/ABS—quantum efficiency of electron transport at t = 0; DIo/ABS—quantum efficiency of energy dissipation (at t = 0); ETo/TRo—electron transport flux per trapping flux in PS II.

**Table 3:** Total phenolic (TP) and organic acids content in the rhizosphere of *P. nigra* L. × *P. maximowiczii* hybrid biomass after 4 weeks of growth in soil enriched with selected metal salts.

Compound [µg g^−1^ DW]	Control	Treatment				
		Pb	Zn	Cu	Al	Ni
TP	31.20 ^a^ ± 2.53	21.24 ^cd^ ± 0.66	21.75 ^b–d^ ± 0.45	24.55 ^b^ ± 0.41	19.49 ^d^ ± 0.51	24.02 ^bc^ ± 0.19
Citric a.	1.46 ^b^ ± 0.06	1.13 ^c^ ± 0.06	1.08 ^c^ ± 0.04	1.57 ^b^ ± 0.12	2.71 ^a^ ± 0.03	0.39 ^d^ ± 0.01
Succinic a.	2.11 ^c^ ± 0.05	2.13 ^c^ ± 0.21	1.70 ^d^ ± 0.06	6.75 ^a^ ± 0.34	4.91 ^b^ ± 0.21	1.00 ^e^ ± 0.05

n = 6; identical superscripts (a, b, c, …) denote non-significant differences between means in rows according to the post hoc Newman–Keuls test; a.—acid.

**Table 4:** Total phenolic (TP) content, phenolic composition and low-molecular-weight organic acids (LMWOAs) content in roots of *P. nigra* L. × *P. maximowiczii* hybrid biomass after 4 weeks of growth in soil enriched with selected metal salts.

Compound	Control	Treatment				
		Pb	Zn	Cu	Al	Ni
TP [mg g^−1^ GAE FW]	0.12 ^d^ ± 0.004	0.13 ^c^ ± 0.002	0.12 ^d^ ± 0.002	0.19 ^a^ ± 0.004	0.14 ^b^ ± 0.004	0.12 ^d^ ± 0.001
Phenolic profile [µg g^−1^ FW]:						
Gallic a.	bdl	bdl	bdl	1.58 ^a^ ± 0.03	bdl	bdl
Vanillic a.	bdl	bdl	bdl	0.29 ^a^ ± 0.003	bdl	bdl
Syringic a.	bdl	1.84 ^a^ ± 0.02	bdl	0.63 ^b^ ± 0.01	bdl	bdl
*p*-Coumaric a.	0.12 ^c^ ± 0.004	0.15 ^b^ ± 0.003	0.10 ^c^ ± 0.003	1.41 ^a^ ± 0.03	0.11 ^c^ ± 0.003	0.10 ^c^ ± 0.003
Ferulic a.	bdl	bdl	bdl	0.25 ^a^ ± 0.005	bdl	bdl
Catechin	0.95 ^a^ ± 0.02	bdl	0.75 ^b^ ± 0.01	bdl	0.31 ^c^ ± 0.006	bdl
Rutin	bdl	0.17 ^a^ ± 0.005	bdl	bdl	bdl	bdl
LMWOAs [µg g^−1^ FW]:						
Malonic a.	0.38 ^b^ ± 0.02	0.34 ^b^ ± 0.02	bdl	7.99 ^a^ ± 0.13	bdl	bdl
Lactic a.	0.18 ^b^ ± 0.01	0.18 ^b^ ± 0.004	0.29 ^a^ ± 0.01	bdl	0.10 ^d^ ± 0.007	0.13 ^c^ ± 0.004
Citric a.	9.69 ^a^ ± 0.42	3.39 ^c^ ± 0.24	1.58 ^d^ ± 0.08	6.09 ^b^ ± 0.68	5.76 ^b^ ± 0.11	bdl
Malic a.	bdl	bdl	bdl	bdl	bdl	0.24 ^a^ ± 0.02
Succinic a.	20.61 ^a^ ± 0.53	7.03 ^c^ ± 0.73	4.53 ^d^ ± 0.06	5.95 ^c^ ± 0.02	11.19 ^b^ ± 0.54	10.08 ^b^ ± 0.78

n = 3; identical superscripts (a, b, c, …) denote non-significant differences between means in rows according to the post hoc Newman–Keuls test; a.—acid; bdl—below detection limit.

**Table 5:** Total phenolic (TP) content, phenolic composition, low-molecular-weight organic acids (LMWOAs) and sugars contents in shoot (leaves and stems) of *P. nigra* L. × *P. maximowiczii* hybrid biomass after 4 weeks of growth in soil enriched with selected metal salts.

Compound	Control	Treatment				
		Pb	Zn	Cu	Al	Ni
TP [mg g^−1^ GAE FW]	6.77 ^c^ ± 0.29	8.46 ^b^ ± 0.30	8.53 ^b^ ± 0.35	8.79 ^ab^ ± 0.20	8.94 ^ab^ ± 0.23	9.22 ^a^ ± 0.34
Phenolic profile [µg g^−1^ FW]:						
Gallic a.	3.87 ^a^ ± 0.24	1.23 ^d^ ± 0.21	1.44 ^d^ ± 0.20	3.01 ^b^ ± 0.43	3.27 ^b^ ± 0.59	1.95 ^c^ ± 0.26
Protocatechuic a.	9.91 ^d^ ± 4.10	15.96 ^d^ ± 2.80	23.47 ^c^ ± 3.96	62.41 ^a^ ± 10.51	26.79 ^c^ ± 4.98	52.89 ^b^ ± 4.37
4-HBA	26.01 ^a^ ± 2.17	18.64 ^b^ ± 2.96	17.53 ^b^ ± 2.36	13.70 ^c^ ± 1.01	14.23 ^c^ ± 1.47	bdl
Sinapic a.	4.20 ^a^ ± 0.27	1.89 ^b^ ± 0.40	2.08 ^b^ ± 0.75	4.46 ^a^ ± 0.24	4.20 ^a^ ± 0.46	4.69 ^a^ ± 0.94
Vanillic a.	3.95 ^bc^ ± 0.28	3.34 ^c^ ± 0.55	4.43 ^bc^ ± 0.46	5.55 ^b^ ± 0.41	9.58 ^a^ ± 1.63	8.84 ^a^ ± 2.09
Syringic a.	8.08 ^d^ ± 0.46	25.52 ^c^ ± 6.42	18.89 ^c^ ± 4.15	50.99 ^a^ ± 8.00	34.45 ^b^ ± 3.19	49.14 ^a^ ± 9.57
*t*-Cinnamic a.	19.82 ^c^ ± 1.41	27.87 ^c^ ± 6.16	23.94 ^c^ ± 4.24	60.73 ^a^ ± 9.18	42.40 ^b^ ± 7.90	42.19 ^b^ ± 8.38
2,5-DHBA	65.37 ^ab^ ± 7.21	24.50 ^c^ ± 5.95	14.38 ^c^ ± 1.74	78.02 ^a^ ± 22.14	52.69 ^b^ ± 6.87	bdl
Chlorogenic a.	12.80 ^b^ ± 1.20	4.73 ^c^ ± 1.51	3.36 ^c^ ± 0.52	12.66 ^b^ ± 2.33	16.27 ^b^ ± 2.64	33.25 ^a^ ± 9.80
Caffeic acid	3.85 ^c^ ± 0.52	2.64 ^c^ ± 0.88	3.10 ^c^ ± 1.05	15.44 ^b^ ± 2.97	11.01 ^b^ ± 4.75	24.16 ^a^ ± 6.26
*p*-Coumaric a.	14.03 ^b^ ± 1.00	8.29 ^c^ ± 2.23	4.77 ^c^ ± 1.79	15.00 ^b^ ± 4.49	14.78 ^b^ ± 1.93	20.86 ^a^ ± 5.61
Ferulic a.	20.11 ^a^ ± 2.90	5.39 ^c^ ± 1.75	4.83 ^c^ ± 1.17	17.08 ^ab^ ± 5.57	14.41 ^b^ ± 1.80	15.63 ^ab^ ± 3.61
Rutin	56.37 ^c^ ± 5.30	22.96 ^c^ ± 4.67	38.63 ^c^ ± 4.22	124.4 ^b^ ± 32.77	149.1 ^b^ ± 19.60	210.4 ^a^ ± 39.86
Catechin	9.02 ^d^ ± 0.68	2.13 ^e^ ± 0.33	1.79 ^e^ ± 0.29	12.55 ^c^ ± 2.75	19.18 ^a^ ± 3.69	15.92 ^b^ ± 7.58
Quercetin	29.65 ^b^ ± 3.87	11.57 ^c^ ± 3.46	22.82 ^bc^ ± 6.80	35.19 ^b^ ± 8.08	40.18 ^b^ ± 6.51	68.86 ^a^ ± 27.72
Kaempferol	5.35 ^c^ ± 1.00	6.29 ^c^ ± 1.63	21.26 ^b^ ± 3.64	32.24 ^a^ ± 4.69	19.43 ^b^ ± 6.52	26.20 ^b^ ± 5.37
Salicylic a.	0.31 ^c^ ± 0.07	0.87 ^b^ ± 0.28	0.77 ^b^ ± 0.14	1.21 ^a^ ± 0.35	0.42 ^c^ ± 0.08	0.26 ^c^ ± 0.06
LMWOAs [µg g^−1^ FW]:						
Oxalic a.	3.60 ^b^ ± 0.84	2.07 ^c^ ± 0.70	0.98 ^d^ ± 0.32	3.05 ^b^ ± 0.57	5.61 ^a^ ± 0.98	3.99 ^b^ ± 0.66
Malonic a.	137.0 ^b^ ± 51.21	13.84 ^c^ ± 4.38	25.92 ^c^ ± 13.16	60.30 ^c^ ± 13.47	7.43 ^c^ ± 0.95	296.5 ^a^ ± 63.49
Lactic a.	7.49 ^ab^ ± 2.91	11.17 ^a^ ± 5.07	9.15 ^ab^ ± 1.76	12.02 ^a^ ± 2.78	5.24 ^b^ ± 1.36	9.39 ^ab^ ± 0.68
Citric a.	2787 ^a^ ± 591.6	1071 ^c^ ± 414.9	1113 ^c^ ± 351.7	2853 ^a^ ± 441.8	1764 ^b^ ± 149.0	2767 ^a^ ± 439.6
Acetic a.	13.35 ^a^ ± 4.21	6.13 ^b^ ± 2.40	4.69 ^b^ ± 1.75	13.87 ^a^ ± 4.22	4.27 ^b^ ± 1.81	6.21 ^b^ ± 1.28
Malic a.	bdl	16.94 ^c^ ± 3.50	32.69 ^b^ ± 5.46	41.08 ^a^ ± 5.02	43.23 ^a^ ± 8.06	13.33 ^c^ ± 2.38
Succinic a.	2130 ^ab^ ± 444.2	1682 ^bc^ ± 772.2	1538 ^bc^ ± 307.5	2544 ^a^ ± 439.1	1206 ^c^ ± 334.2	1934 ^a–c^ ± 459.0
Fumaric a.	1.39 ^a^ ± 0.18	0.81 ^b^ ± 0.24	0.70 ^b^ ± 0.14	1.47 ^a^ ± 0.34	0.86 ^b^ ± 0.19	1.71 ^a^ ± 0.29
Sugars [mg g^−1^ FW]:						
Fructose	1.74 ^c^ ± 0.15	3.40 ^a^ ± 0.50	3.13 ^a^ ± 0.52	2.43 ^b^ ± 0.36	3.66 ^a^ ± 0.40	3.05 ^a^ ± 0.55
Glucose	2.36 ^b^ ± 0.26	2.60 ^b^ ± 0.43	1.77 ^c^ ± 0.15	3.34 ^a^ ± 0.53	1.63 ^c^ ± 0.28	2.09 ^bc^ ± 0.15
Sucrose	2.52 ^d^ ± 0.25	4.63 ^c^ ± 0.90	4.14 ^c^ ± 0.40	4.46 ^c^ ± 0.75	7.61 ^a^ ± 0.76	6.09 ^b^ ± 0.79

n = 6; identical superscripts (a, b, c, …) denote non-significant differences between means in rows according to the post hoc Newman–Keuls test; a.—acid; bdl—below detection limit.

## Data Availability

The dataset is available upon request from the authors.

## Supplementary Materials

The following supporting information can be downloaded at: https://www.mdpi.com/article/10.3390/ijms252312520/s1.
