# Supplementary material for: Early Response of the Populus nigra L. × P. maximowiczii Hybrid to Soil Enrichment with Metals
Source: Int J Mol Sci. 2024 Nov 21;25(23):12520. doi: 10.3390/ijms252312520 (PMC11641318; doi:10.3390/ijms252312520)
Supplement: Supplementary file 1 [file ijms-25-12520-s001.zip › ijms-3295338-Suplementary Materials.pdf]

Table S1. The recovery of certified reference materials (CRMs) representing wood (NIST SRM 2790) and leaves (INCT−TL−1) used in quality control.

Elements	NIST SRM 2790			INCT–TL–1		
	**Certified (mg kg^{-1})**	**Detected (mg kg^{-1})**	**Recovery (%)**	**Certified (mg kg^{-1})**	**Detected (mg kg^{-1})**	**Recovery (%)**
Al	92.06 [a]	85.2	93	2290	1840	80
Ca	×	2400	–	5820	6690	115
Cu	1.545 [a]	1.69	109	20.4	17.7	87
Fe	82.9	83.2	100	432 [b]	357	83
K	1040	1210	116	17000	14300	84
Mg	189	158	84	2240	1790	80
Na	26	23.1	89	24.7	21.2	86
Ni	×	0.971	–	6.12	4.90	80
Pb	×	1.29	–	1.78	1.69	95
Zn	9.26	8.51	92	34.7	25.9	75

[a] – reference value; [b] – information value; × – non–certified value.

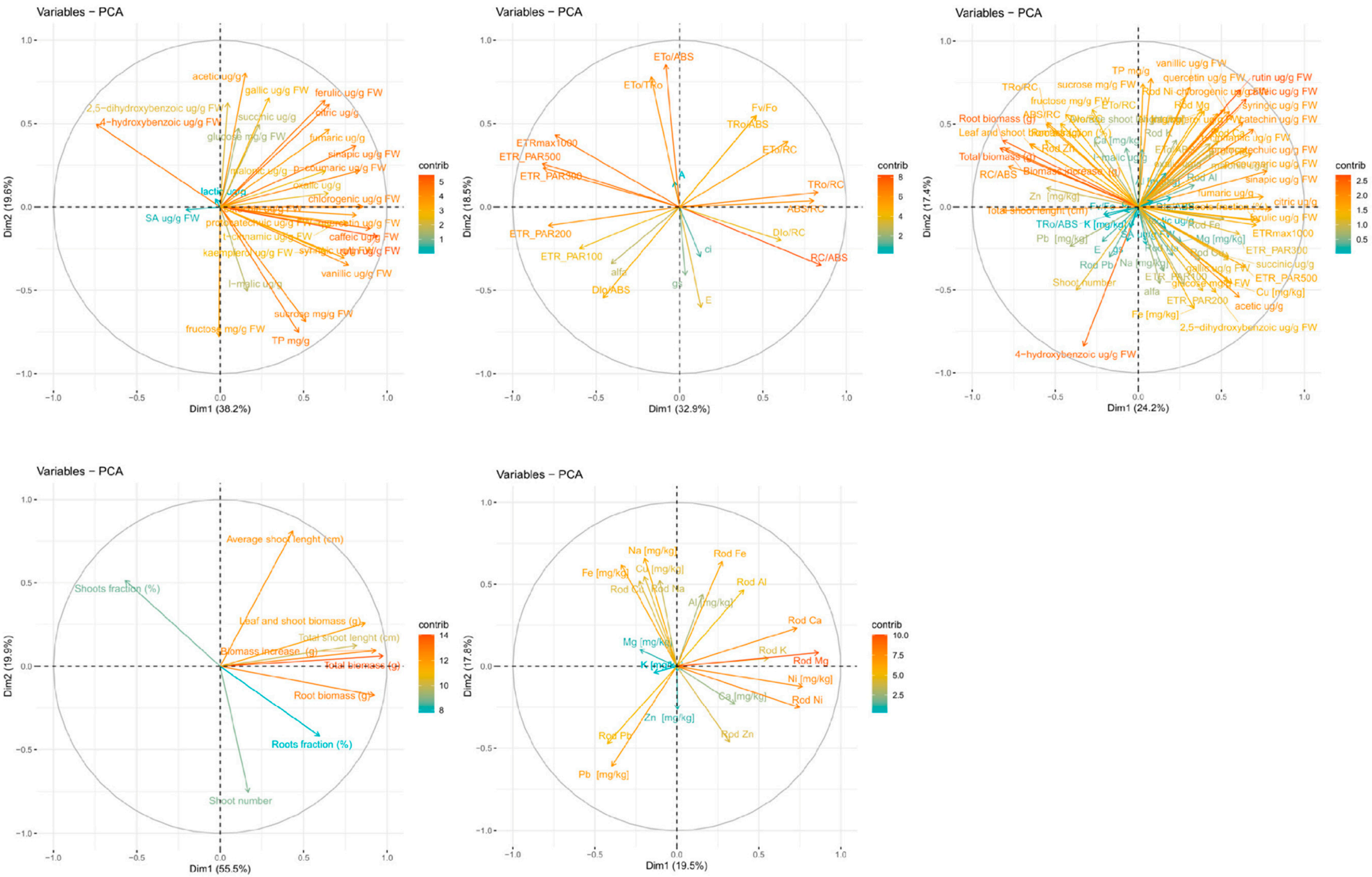

Figure S1. The biplots showing particular variables employed for the PCA analysis and their contribution to the variance explained by the first and second axes.
